# KCa3.1 Promotes Proinflammatory Exosome Secretion by Activating AKT/Rab27a in Atrial Myocytes during Rapid Pacing

**DOI:** 10.1155/2023/3939360

**Published:** 2023-03-30

**Authors:** Dishiwen Liu, Huiyu Chen, Yuntao Fu, Yajun Yao, Shanqing He, Youcheng Wang, Zhen Cao, Xuewen Wang, Mei Yang, Qingyan Zhao

**Affiliations:** ^1^Department of Cardiology, Renmin Hospital of Wuhan University, Wuhan 430060, China; ^2^Cardiovascular Research Institute, Wuhan University, Wuhan 430060, China; ^3^Hubei Key Laboratory of Cardiology, Wuhan 430060, China

## Abstract

**Purpose:**

The aim of this study was to investigate the role of the medium-conductance calcium-activated potassium channel (KCNN4, KCa3.1) in the secretion of proinflammatory exosomes by atrial myocytes.

**Methods:**

Eighteen beagles were randomly divided into the sham group (*n* = 6), pacing group (*n* = 6), and pacing+TRAM-34 group (*n* = 6). Electrophysiological data, such as the effective refractory period, atrial fibrillation (AF) induction, and AF duration, were collected by programmed stimulation. Atrial tissues were subjected to hematoxylin and eosin, Masson's trichrome, and immunofluorescence staining. The expression of KCa3.1 and Rab27a was assessed by immunohistochemistry and western blotting. The downstream signaling pathways involved in KCa3.1 were examined by rapid pacing or overexpressing KCNN4 in HL-1 cells.

**Results:**

Atrial rapid pacing significantly induced electrical remodeling, inflammation, fibrosis, and exosome secretion in the canine atrium, while TRAM-34 (KCa3.1 blocker) inhibited these changes. Compared with those in control HL-1 cells, the levels of exosome markers and inflammatory factors were increased in pacing HL-1 cells. Furthermore, the levels of CD68 and iNOS in macrophages incubated with exosomes derived from HL-1 cells were higher in the pacing-exo group than in the control group. More importantly, KCa3.1 regulated exosome secretion through the AKT/Rab27a signaling pathway. Similarly, inhibiting the downstream signaling pathway of KCa3.1 significantly inhibited exosome secretion.

**Conclusions:**

KCa3.1 promotes proinflammatory exosome secretion through the AKT/Rab27a signaling pathway. Inhibiting the KCa3.1/AKT/Rab27a signaling pathway reduces myocardial tissue structural remodeling in AF.

## 1. Introduction

Atrial fibrillation (AF) is a major public health problem worldwide and is associated with high morbidity and mortality [1]. At present, there is much evidence to show that AF is closely related to electrical remodeling, structural remodeling, and neural remodeling [2–4]. However, the mechanism of electrical-substrate remodeling in AF induction and maintenance has not been fully elucidated.

Atrial myocytes are the main cellular components of the atria and are responsible for contractile and secretory functions. In pathological states, atrial myocytes influence the surrounding microenvironment by secreting relevant substances. Sun et al. found that macrophages polarized toward a proinflammatory phenotype in the right atrial appendage of patients with AF [5]. However, the researchers did not investigate which substances secreted by atrial myocytes caused macrophage polarization. In fact, tachycardia in atrial myocytes induced the paracrine secretion of angiotensin (Ang) II, reactive oxygen species, and TGF-*β*1 [6]. However, whether atrial myocytes affect peripheral cells through other forms of secretion has been poorly studied.

The medium-conductance calcium-activated potassium channel (KCNN4, KCa3.1) is a member of the calcium-activated potassium channel family and has been intensively studied due to its wide range of functions, including proliferation, differentiation, and arrhythmogenesis. In cardiomyocytes that were induced by pluripotent stem cells from patients with catecholamine-sensitive polymorphic ventricular tachycardia, the KCa3.1 blocker significantly reduced the delay after depolarizations and arrhythmic Ca^2+^ transients [7]. Moreover, our previous research showed that KCa3.1 was involved in early depolarization and triggering, and the KCa3.1 inhibitor TRAM-34 significantly inhibited electrical remodeling and completely inhibited the induction of acute AF [8]. Our recent study in canines further showed that TRAM-34 abrogated atrial fibrosis and inflammation in atrial rapid pacing [9]. However, the arrhythmogenic effects of KCa3.1 that contribute to the progression of AF toward permanence are unclear. The mechanism of KCa3.1 in structural remodeling and ion remodeling should be further investigated.

Exosomes are extracellular vesicles that carry a variety of signaling molecules from donor cells (DNA, mRNA, microRNA, lncRNA, and proteins) [10]. Our previous study investigated the relationship between AF and exosomes through bioinformatic analysis. We verified that cargo-carrying exosomes secreted by fibroblasts in AF facilitated atrial myocyte ferroptosis [11]. However, it is still unclear how atrial myocytes (as initiating factors) promote the local inflammatory response based on electrical remodeling. Therefore, we hypothesize that exosomes secreted by atrial myocytes play a crucial role in the delivery of inflammatory signals.

Rab27a, which is a member of the RAS oncogene family, affects exosome secretion by regulating the docking and fusion of multiple vesicle bodies with the cell membrane [12]. Inhibiting Rab27a predominantly downregulates the secretion of matrix metalloprotein 9 and platelet-derived growth factor A [13]. Studies have shown that PI3K/AKT affects Rab27a-related vesicle secretion by regulating the phosphorylation of the Rab27a-binding protein JFC1 [14]. In another study, Huang et al. suggested that KCa3.1 was closely related to the PI3K/AKT signaling pathway [15]. Based on these studies, we hypothesized that KCa3.1 could regulate Rab27a through the AKT signaling pathway to affect exosome secretion. The purpose of this study was to examine whether abnormal expression of KCa3.1 leads to exosome secretion and the downstream signaling pathways involved.

## 2. Materials and Methods

This study was approved by the Animal Studies Subcommittee of our Institutional Review Board and was performed in accordance with the guidelines of the National Institutes of Health for the care and use of laboratory animals.

### 2.1. Animal Model Preparation

The rapid atrial pacing canine model was prepared as previously described [11]. Eighteen beagles were randomly divided into the sham group (*n* = 6), pacing group (*n* = 6), and pacing+TRAM-34 group (*n* = 6). Three days after the pacemaker was implanted, the pacing+TRAM-34 group was given an intravenous injection of TRAM-34 (10 mg/kg/d, tid, MCE, United States). The pacing group and the pacing+TRAM-34 group were paced at 450 bpm for seven days.

### 2.2. Electrophysiological Measurement

After seven consecutive days of intravenous administration of TRAM-34, the canines were anesthetized, a bilateral thoracotomy was performed, and procedural stimulation was performed after placing multilead electrodes in various parts of the atrium. Briefly, we measured the atrial effective refractory period (ERP) and the inducibility and duration of AF [11].

### 2.3. Histopathology

The canines were subjected to electrophysiological testing after seven days of continuous rapid pacing and then were anesthetized and executed, followed by the sampling of atrial tissue. Paraffin embedding was performed after fixation using paraformaldehyde. For histological analysis, canine atrial tissues were sectioned into 5 *μ*M thick slices. The sections were then stained with hematoxylin and eosin and Masson's trichrome to assess inflammatory infiltration and collagen deposition, respectively. To determine the expression of KCa3.1, CD68, CD81, cTnI, iNOS, and Rab27a, the sections were incubated with KCa3.1 (Proteintech, China; 1 : 100), CD68 (Servicebio, China; 1 : 200), CD81 (Proteintech, China; 1 : 200), cTnI (Affinity, China; 1 : 200), iNOS (Servicebio, China; 1 : 200), and Rab27a antibodies (Proteintech, China; 1 : 300). Immunohistochemistry and immunofluorescence images were captured with a fluoroscope and analyzed by ImageJ.

### 2.4. Cell Culture and Treatments

The mouse atrial HL-1 cell line (Procell, China) or mouse RAW264.7 macrophage cell line (Wuhan University, China) was cultured in F12/DMEM (Gibco, United States) or DMEM (High glucose, Gibco, United States) containing 10% fetal bovine serum and 1% penicillin/streptomycin. The rapid-pacing HL-1 cell model was prepared as previously described [11]. Briefly, HL-1 cells were paced by electric field stimulation (600 times/min) at an intensity of 1.5 V/cm with continuous stimulation for 48 h. In the different treatment groups, HL-1 cells were treated with TRAM-34 (20 *μ*M), BAPTA (20 *μ*M, selective chelator for calcium, MCE, United States), GSK690693 (20 *μ*M, ATP-competitive pan-AKT inhibitor, MCE, United States), or SC79 (specific AKT activator, MCE, United States). Follow-up assays were then performed.

### 2.5. Lentivirus and Cell Infection

KCNN4 and GFP lentiviruses were produced by GeneChem (Shanghai, China). HL-1 cells were infected with lenti-KCNN4 and lenti-GFP at a multiplicity of infection of 30 MOI for 24 h. Subsequently, pressure screening was performed using puromycin at 3 *μ*g/ml for 72 h. Then, the dose of puromycin was adjusted to 1 *μ*g/ml for routine pressure screening. The expression of KCa3.1 was evaluated by western blotting.

### 2.6. siRNA Transfection

siRNA duplexes targeting Rab27a and a negative control siRNA were designed and synthesized by GenePharma (Shanghai, China). The Rab27a siRNA sequences were 5′-CGGAUCAGUUAAGUGAAGAAA-3′ and antisense 5′-UUCUCCGAACGUGUCACGU-3′. The cells were grown to 60-80% confluence in 6-well dishes. For each well, serum-free F12/DMEM was mixed separately with siRNA and Lipofectamine 6000 (Beyotime, China) for 5 min. Then, the siRNA and Lipofectamine 6000 were mixed gently and incubated at room temperature for 10 min. Subsequently, the diluted siRNA/Lipofectamine 6000 complex was added to the 6-well dish and incubated for 5 h, after which the complex was replaced with a normal cell culture medium.

### 2.7. Exosome Isolation

Cell debris and large vesicles were separated by differential centrifugation at 300 × g, 3000 × g, and 10000 × g. Subsequently, the supernatant was concentrated using ultrafiltration tubes (Millipore, 10000 MW, United States) at 4000 × g. Finally, the supernatant was ultracentrifuged at 120000 × g for 90 min to obtain exosomes. All procedures were carried out at 4°C. Exosomes were resuspended in PBS and stored at -80°C for subsequent analysis.

### 2.8. Transmission Electron Microscopy

The exosome suspension was fixed with 4% paraformaldehyde. Exosomes were then adsorbed onto formvar-carbon-coated copper grids. Subsequently, the grids were rinsed in PBS and negatively stained with 2% uranyl acetate for 5 min at room temperature and then photographed with a JEM-1100 transmission electron microscope at an accelerating voltage of 80 kV.

### 2.9. Western Blotting

We extracted proteins from 30 mg of canine atrial tissue or cultured cells. The protein concentration was determined by a BCA Protein Assay Kit (Aspen, as1086) according to the manufacturer's instructions. Proteins were separated on a 10% SDS-polyacrylamide gel and transferred to a 0.45 *μ*M PVDF membrane by semidry transfer. The PVDF membranes were then blocked with 1% polyvinylpyrrolidone-40 and 0.05% Tween-20 for 30 minutes. The expression of target proteins was determined by incubating the membranes with the following primary antibodies overnight at 4°C: GAPDH (Servicebio, China, 1 : 1000), CD81 (Abmart, China, 1 : 1000), Rab27a (Servicebio, China, 1 : 1000), KCa3.1 (Proteintech, China, 1 : 1000), TSG101 (Servicebio, China, 1 : 1000), AKT (Servicebio, China, 1 : 1000), and p-AKT (Abmart, China, 1 : 1000). Visualization was performed on a chemiluminescence system after incubation with horseradish peroxidase-conjugated secondary antibodies (Proteintech, China, 1 : 3000) at room temperature.

### 2.10. Enzyme-Linked Immunosorbent Assay (ELISA)

The production of TNF-*α* (Invitrogen, United States), IL-1*β* (Invitrogen, United States), IL-6 (Invitrogen, United States), and TGF-*β*1 (Lianke, China) was determined using commercial ELISA kits. Samples were prepared and tested according to the manufacturer's instructions.

### 2.11. Quantitative Real-Time PCR

Total RNA was extracted from cells using the TRIzol® reagent (Takara, Japan). Isolated RNA (2 *μ*g) was converted into complementary DNA using the RT First Strand cDNA Synthesis Kit (Servicebio, China). The cDNA templates were amplified by a qRT-PCR system (Applied Biosystem, United States) using SYBR Green PCR Mix (Servicebio, China) with the corresponding primers (Table 1). The 2^-*ΔΔ*Ct^ comparative quantification method was used to analyze the semilog amplification curves, and target gene expression was normalized to GAPDH.

### 2.12. Statistical Analysis

The results are presented as the mean ± SD. Comparisons among the different groups were performed with one-way ANOVA and post hoc Tukey's test. *p* < 0.05 was regarded as statistically significant. All data were analyzed using GraphPad Prism 8 (GraphPad, United States) or SPSS 25.0 (IBM, United States).

## 3. Results

### 3.1. TRAM-34 Inhibits Electrical Remodeling in Canines with Rapid Atrial Pacing

**Figure 1:**
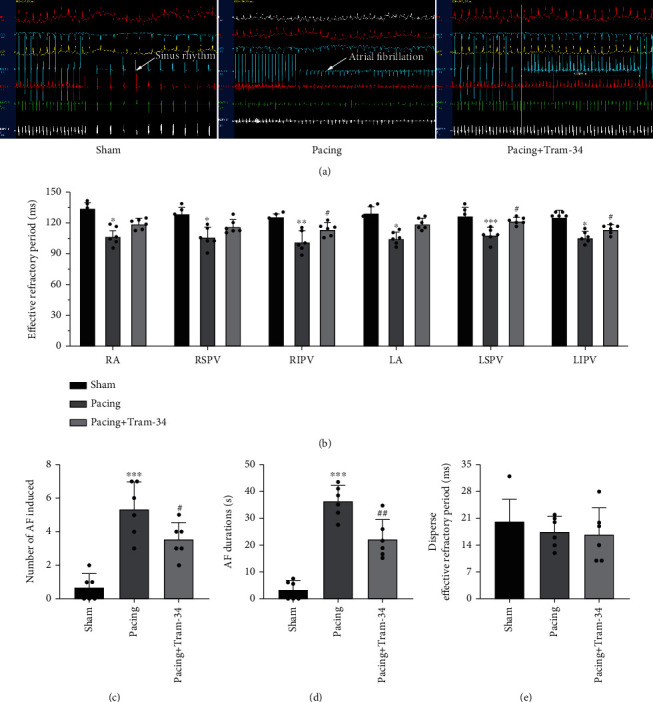
Canine electrophysiological examination during programmed stimulation. (a) The state of atrial fibrillation (AF) could not be induced in the sham group, and the state of AF was induced in the pacing group, while the state of AF was no more than 5 seconds in the pacing+TRAM-34 group. (b) Effective refractory period in different parts of the atrium. TRAM-34 increased the ERP of RIPV, LSPV, and LIPV after rapid atrial pacing. (c) Difference in AF inducibility, as shown by the number of episodes. (d) Difference in mean AF durations. (e) Difference in the dispersed effective refractory period. The data are presented as the mean ± SD of six biological replicates. ^∗^*p* < 0.05, ^∗∗^*p* < 0.01, and ^∗∗∗^*p* < 0.001 vs. sham group; ^#^*p* < 0.05 and ^##^*p* < 0.01 vs. pacing group. Abbreviations: RA: right atrium; RSPV: right superior pulmonary vein; RIPV: right inferior pulmonary vein; LA: left atrium; LSPV: left superior pulmonary vein; LIPV: left inferior pulmonary vein; ERP: effective refractory period.

Compared with that in the sham group, the ERP at the recording sites was significantly shortened in the pacing group, and RIPV, LSPV, and LIPV were increased by treatment with TRAM-34 (113.7 ± 5.8 vs. 101.2 ± 10.4 ms in the RIPV, *p* < 0.05; 121.3 ± 3.5 vs. 109.3 ± 8.0 ms in the LSPV, *p* < 0.05; 118.0 ± 6.1 vs. 105.7 ± 6.3 ms in the LIPV) (Figure 1(b)). AF was more easily induced in the pacing group, and the administration of TRAM-34 reduced AF induction by 1.8 times (3.5 ± 1.0 vs. 5.3 ± 1.6 times, *p* < 0.05) (Figure 1(c)). The effect of TRAM-34 on electrophysiology was also reflected by the shortening of the duration of AF. Compared with that in the pacing group, TRAM-34 reduced the mean duration of AF by at least 10 s (22.2 ± 7.3 vs. 36.3 ± 5.9 s, *p* < 0.01) (Figures 1(a) and 1(d)). We did not observe significant differences in dispersed ERP among the three groups (Figure 1(e)).

### 3.2. TRAM-34 Alleviates Inflammatory Infiltration in Canine Atrial Tissue during Rapid Pacing

**Figure 2:**
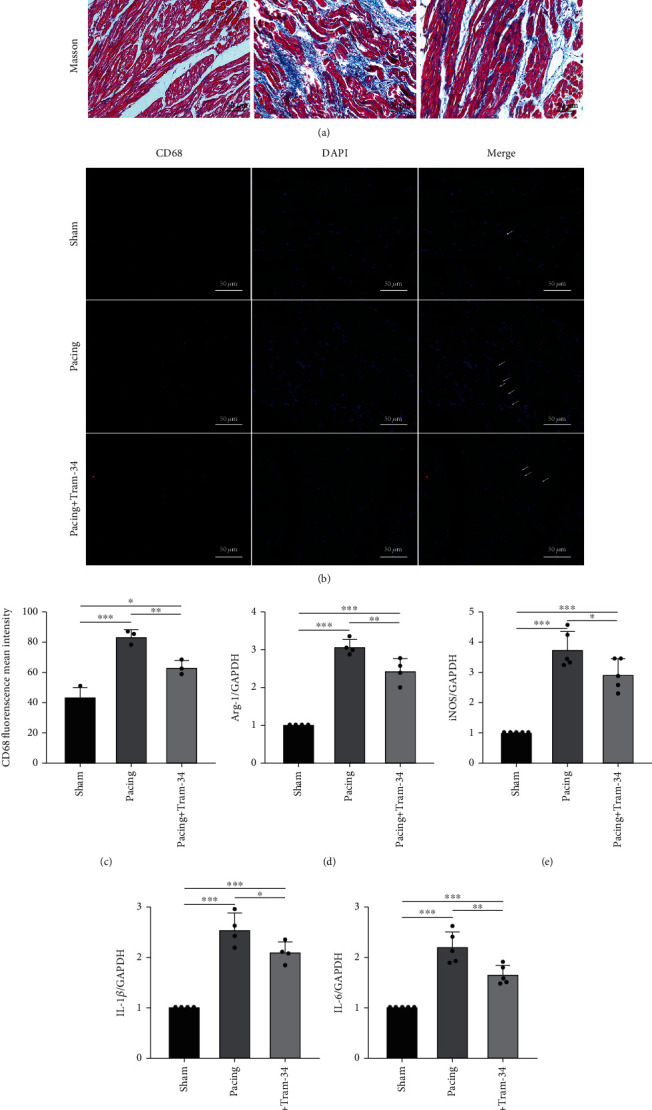
TRAM-34 attenuates inflammation in atrial tissue during rapid pacing. (a) Representative images of inflammation and fibrosis, as reflected by the H&E staining and Masson staining. Black arrows indicate the infiltration of inflammatory cells. (b, c) Representative images of CD68, as reflected by immunofluorescence analysis. White arrows indicate CD68-positive macrophages. (d–g) RT-PCR analysis of Arg-1, iNOS, IL-1*β*, and IL-6 expression normalized to GAPDH in atrial tissue during rapid pacing. The data are presented as the mean ± SD of three to six biological replicates. ^∗^, ^∗∗^, and ^∗∗∗^ indicate *p* < 0.05, 0.01, and 0.001, respectively.

At the histochemical level, we observed high levels of neutrophil and monocyte infiltration in the atrial myocyte interstitium in the pacing group, whereas TRAM-34 significantly reduced inflammatory infiltration. Compared with the sham group, the pacing group had disorganized atrial myocytes, and this effect was improved by TRAM-34 (Figure 2(a)). As shown in Figure 2(a), the deposition of collagen in the pacing group was significantly higher than that in the sham group, and TRAM-34 attenuated the deposition of collagen. To further observe macrophage polarization and migration, we stained CD68, which is a marker of M1 macrophages. Our results demonstrated a significant increase in CD68-positive cells in the pacing group, which was reversed by the administration of TRAM-34 (Figures 2(b) and 2(c)). Furthermore, compared with those in the sham group, the levels of Arg-1 and iNOS, which are markers of M1 macrophage polarization, were predominantly increased at the transcriptional level in the pacing group, and TRAM-34 alleviated this increase (Figures 2(d) and 2(e)). Regarding inflammatory factor expression, IL-1*β* and IL-6 transcript levels were significantly increased in the pacing group, while TRAM-34 was effective in reducing their expression levels (Figures 2(f) and 2(g)). Collectively, these results showed that TRAM-34 had anti-inflammatory effects.

### 3.3. Blocking KCa3.1 Inhibits Exosome Secretion in Rapid-Pacing Canine Atrial Tissue

**Figure 3:**
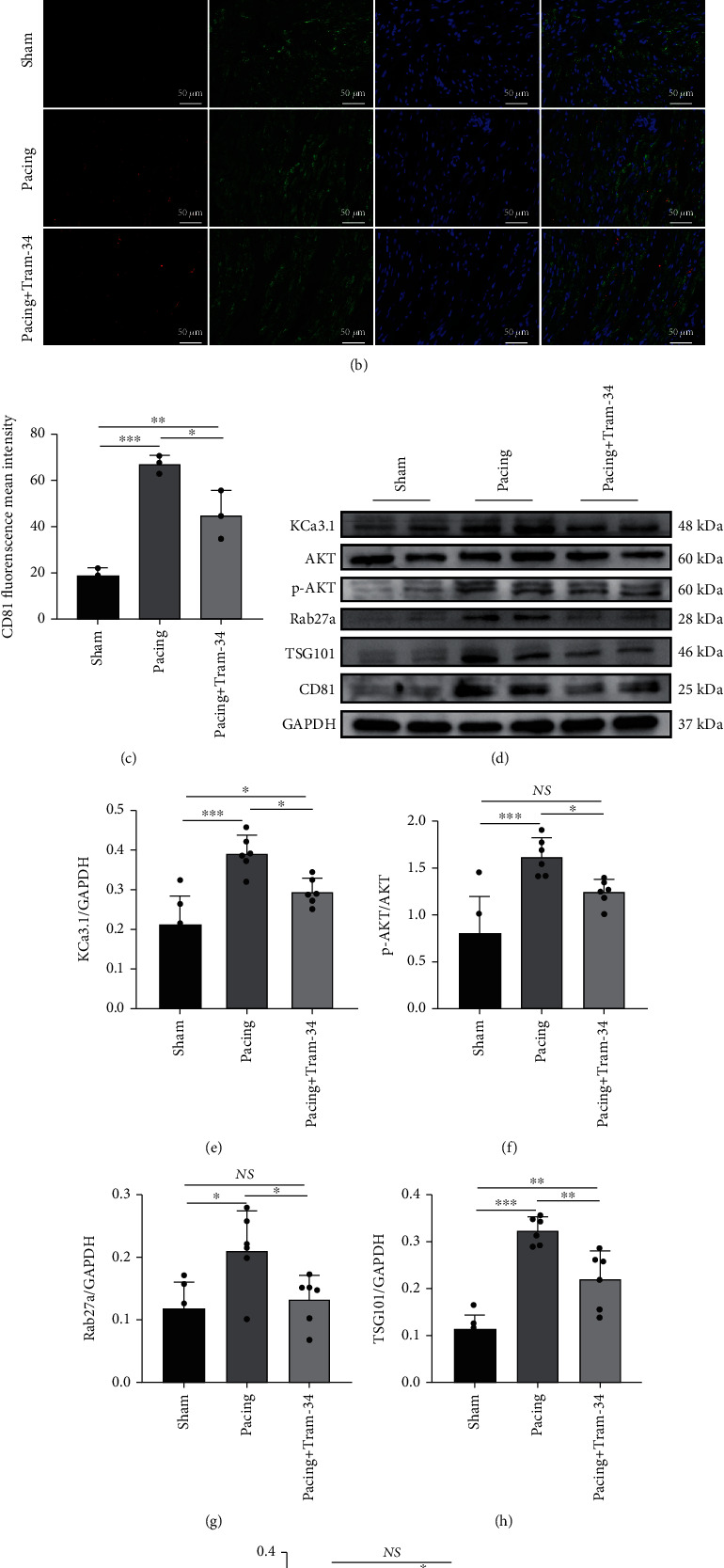
TRAM-34 attenuates the secretion of exosomes in atrial tissue during rapid pacing. (a) Representative images of KCa3.1 and Rab27a, as reflected by immunohistochemistry. (b, c) Representative images of CD81, as reflected by immunofluorescence analysis. (d) Representative gel bands showing the effects of TRAM-34 administration on atrial tissue during rapid pacing. (e–i) Levels of KCa3.1, p-AKT/AKT, Rab27a, TSG101, and CD81. The data are presented as the mean ± SD of three to six biological replicates. ^∗^, ^∗∗^, and ^∗∗∗^ indicate *p* < 0.05, 0.01, and 0.001, respectively.

We examined whether rapid pacing increased exosome secretion and whether the KCa3.1-specific blocker TRAM-34 affected exosome secretion in vivo. Our results showed that KCa3.1 expression was increased in the pacing group, which was supported by the immunoblotting results (Figures 3(d) and 3(e)). In addition, the immunohistochemical results showed darker cell staining in the pacing group (Figure 3(a)). As expected, compared with that in the pacing group, TRAM-34 significantly reduced KCa3.1 expression (Figures 3(a), 3(d), and 3(e)). Rab27a, which is related to exosome secretion, was also significantly increased in the pacing group. Consistent with the change in KCa3.1, the administration of TRAM-34 reduced the expression of Rab27a in the pacing group, which was supported by the immunoblotting and immunohistochemistry results (Figures 3(a), 3(d), and 3(g)).

To further investigate whether exosomes were secreted by atrial myocytes, we double-stained atrial tissue for CD81 and cTnI. Interestingly, the results showed that the distributions of the exosome marker CD81 and the atrial myocyte-specific marker cTnI were almost identical. Additionally, TRAM-34 greatly attenuated the expression of CD81 (Figures 3(b) and 3(c)). In addition, immunoblotting showed that the expression of exosome markers such as CD81 and TSG101 was increased in the pacing group, but these effects were blocked by TRAM-34 (Figures 3(d), 3(h), and 3(i)). Compared with that in the sham group, the expression of phosphorylated AKT was significantly increased in the pacing group, and TRAM-34 reversed this change (Figures 3(d) and 3(f)). Taken together, these results suggested that TRAM-34 could block exosome secretion through the AKT/Rab27a signaling pathway.

### 3.4. The Effects of Rapid Pacing on Exosome Secretion and Inflammation in HL-1 Cells

**Figure 4:**
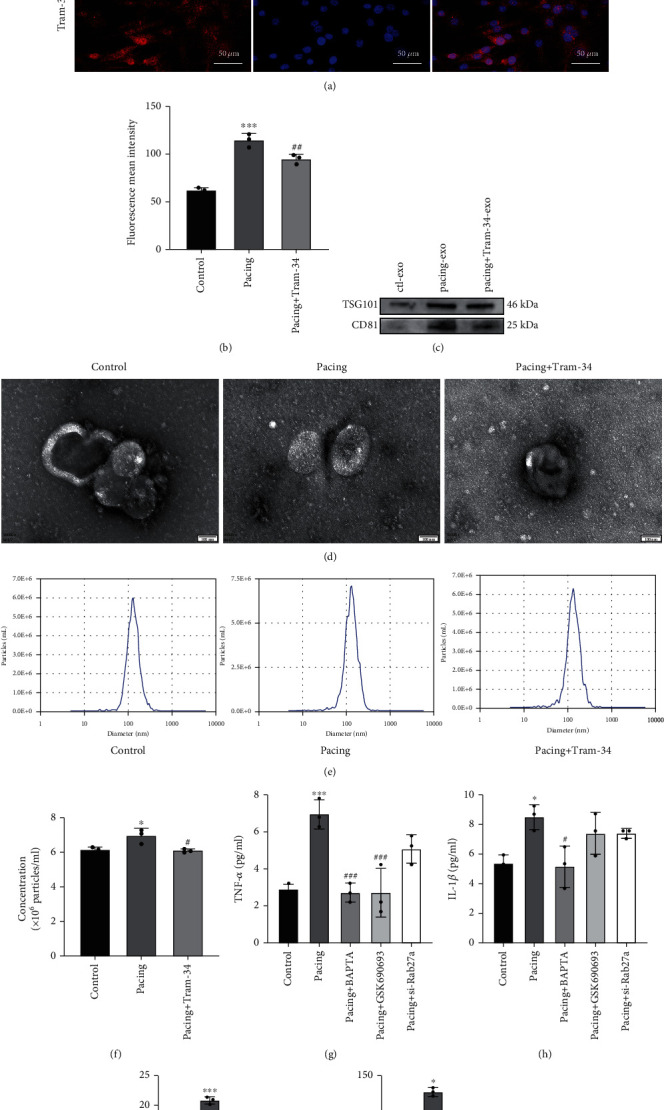
Rapid pacing stimulates HL-1 cell secretion. (a, b) Representative images of KCa3.1, as reflected by immunofluorescence analysis. (c) The characteristic markers CD81 and TSG101 in isolated exosomes were detected by immunoblotting. (d) Morphology of isolated exosomes from HL-1 cells, as examined by transmission electron microscopy. (e, f) Mean exosome diameter and concentration, as shown by the Zeta View System. (g–j) TNF-*α*, IL-*β*, IL-6, and TGF-*β*1 concentrations in HL-1 cell supernatants. The exosomes were extracted from the same volume of supernatant from cells of equal density. The data are presented as the mean ± SD of three biological replicates. ^∗^*p* < 0.05 and ^∗∗∗^*p* < 0.001 vs. control group; ^#^*p* < 0.05, ^##^*p* < 0.01, and ^###^*p* < 0.001 vs. pacing group.

To further examine the specific mechanisms of KCa3.1 in AF, we paced HL-1 cells for 48 h. Immunofluorescence analysis showed that KCa3.1 expression in the pacing group was significantly higher than that in the control group, and TRAM-34 reduced the expression of KCa3.1 (Figures 4(a) and 4(b)). To verify the expression of exosome markers, we extracted HL-1 cell exosomes from equalized cell density and equal amounts of supernatant for immunoblotting. Our results showed that the extracted exosomes all expressed CD81 and TSG101, and the markers were higher in the pacing-exo group while TRAM-34 reduced their expression levels (Figure 4(c)). To qualitatively and quantitatively examine the effects of rapid pacing on exosome secretion, we extracted exosomes and examined their morphology and quantity. Transmission electron microscopy indicated that there were no obvious changes in exosome morphology (Figure 4(d)). Nanoparticle tracking analysis demonstrated that the vesicles were between 33 and 230 nm in diameter, most of which were 90-190 nm. After rapid pacing, the concentration of cellular supernatant exosomes increased to 6.97 ± 0.42 × 10^6^ particles/ml from 6.17 ± 0.15 × 10^6^ particles/ml, and the concentration was decreased to 6.10 ± 0.10 × 10^6^ particles/ml by TRAM-34 treatment (Figures 4(e) and 4(f)).

The levels of TNF-*α*, IL-1*β*, IL-6, and TGF-*β*1 in cell supernatants were significantly higher in the pacing group than in the control group (Figures 4(g)–4(j)). Furthermore, compared with those in the pacing group, BAPTA decreased the levels of TNF-*α*, IL-1*β*, IL-6, and TGF-*β*1 (Figures 4(g)–4(j)), while the AKT inhibitor GSK690693 decreased TNF-*α*, IL-6, and TGF-*β*1 levels (Figures 4(g), 4(i), and 4(j)), and si-Rab27a decreased IL-6 and TGF-*β*1 levels (Figures 4(i) and 4(j)). Taken together, these results suggested that blocking the KCa3.1/AKT/Rab27a signaling pathway could reduce exosome secretion and exocrine functions by HL-1 cells.

### 3.5. The Effects of Rapid-Pacing HL-1 Cell-Derived Exosomes on Macrophage Polarization

**Figure 5:**
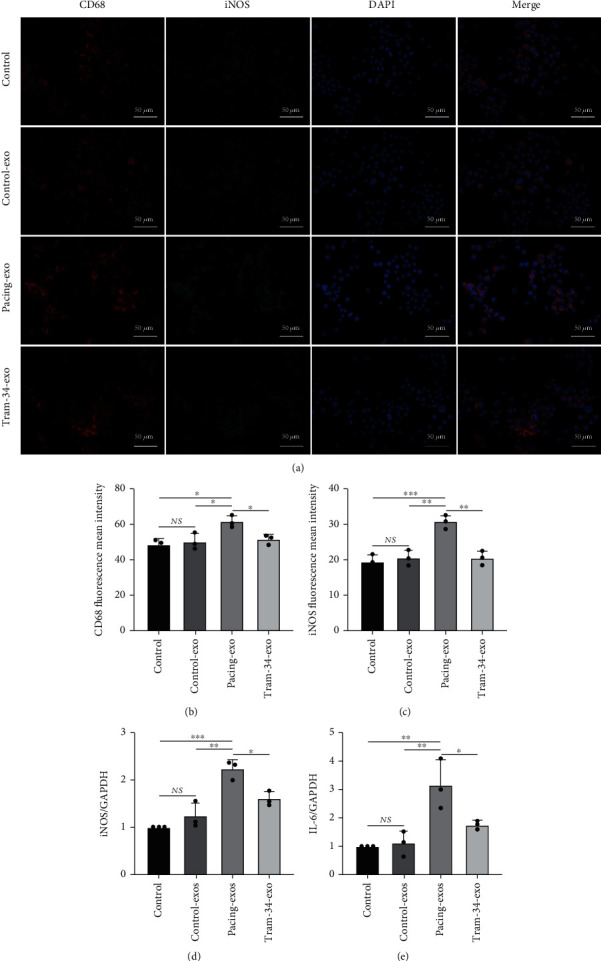
Exosomes derived from rapid-pacing HL-1 cells promote macrophage polarization. (a–c) Representative images of CD68 and iNOS, as reflected by immunofluorescence analysis. (d, e) RT-PCR analysis of iNOS and IL-6 expression normalized to GAPDH in macrophages. The data are presented as the mean ± SD of three biological replicates. ^∗^, ^∗∗^, and ^∗∗∗^ indicate *p* < 0.05, 0.01, and 0.001, respectively.

We incubated HL-1 cell-derived exosomes with macrophages and found that these exosomes had proinflammatory effects. Compared with those in the control-exo group, immunofluorescence analysis showed that CD68 and iNOS levels in the pacing-exo group were significantly increased, whereas TRAM-34 altered this proinflammatory property of exosomes (Figures 5(a)–5(c)). Moreover, RT–PCR showed that iNOS and IL-6 levels were significantly increased in the pacing-exo group (Figures 5(d) and 5(e)). These results suggest that exosomes secreted by rapid-pacing atrial myocytes promote macrophage polarization toward the M1 phenotype.

### 3.6. Blocking Intracellular Calcium Reduces AKT Activation

**Figure 6:**
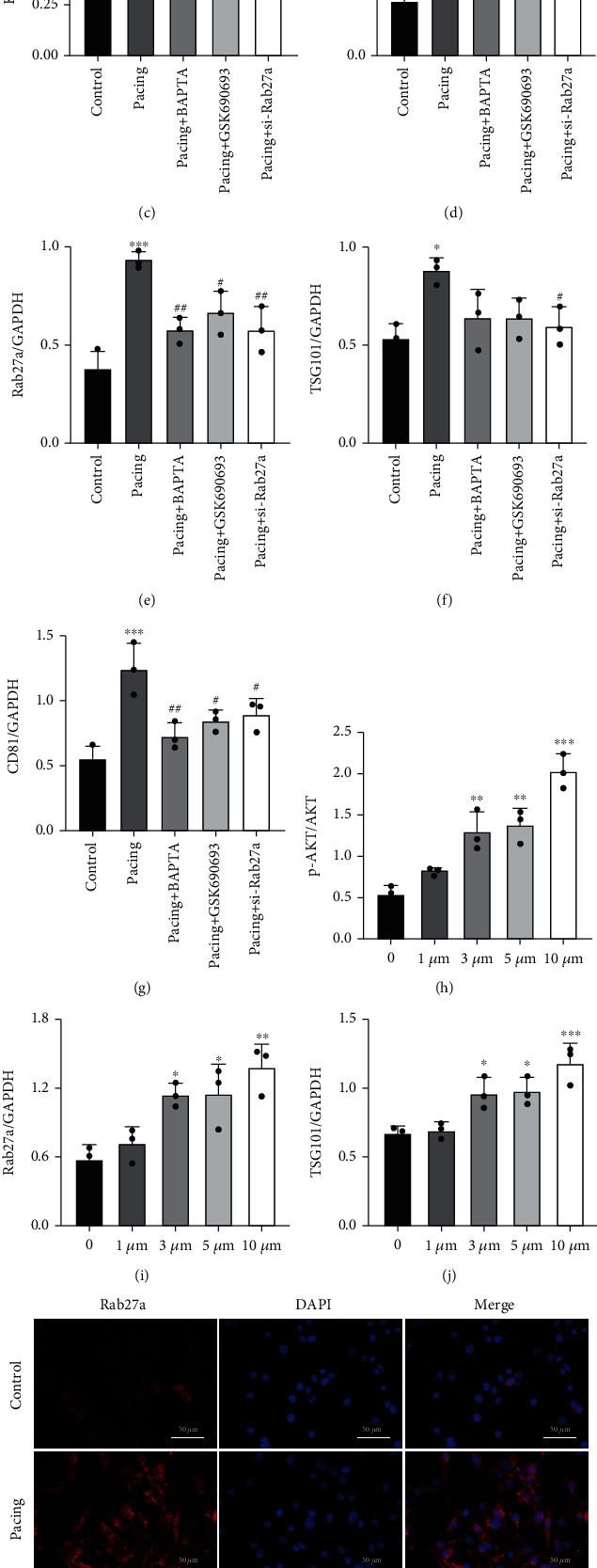
Rapid pacing activates the KCa3.1/AKT/Rab27a signaling pathway. (a) Representative gel bands showing the effects of BAPTA, GSK690693, and si-Rab27a on rapid-pacing HL-1 cells. (b) Representative gel bands showing the effects of different concentrations of SC79. (c–g) The levels of KCa3.1, p-AKT/AKT, Rab27a, TSG101, and CD81 after treatment with BAPTA, GSK690693, and si-Rab27a. (h–j, l) The levels of p-AKT/AKT, Rab27a, TSG101, and CD81 after treatment with different concentrations of SC79. (k, m) Representative images of Rab27a after treatment with BAPTA, GSK690693, and si-Rab27a, as reflected by immunofluorescence analysis. The data are presented as the mean ± SD of three biological replicates. ^∗^*p* < 0.05, ^∗∗^*p* < 0.01, and ^∗∗∗^*p* < 0.001 vs. control group; ^#^*p* < 0.05 and ^##^*p* < 0.01 vs. pacing group.

To determine whether KCa3.1 affects AKT and downstream signaling via calcium ions, we used the calcium chelators BAPTA and GSK690693 and si-Rab27a to intervene in rapid-pacing HL-1 cells. Our results demonstrated that the expression of KCa3.1 did not significantly change (Figures 6(a) and 6(c)), indicating that blocking the downstream cascade did not affect the expression of KCa3.1. By intervening with intracellular calcium with BAPTA, we found that the phosphorylation level of AKT was significantly reduced in the pacing group (Figures 6(a) and 6(d)). In addition, the downstream signaling factors, including Rab27a, TSG101, and CD81, were significantly increased in the pacing group, while BAPTA alleviated those changes (Figures 6(a) and 6(e)–6(g)).

### 3.7. AKT Activation Affects the Expression of Rab27a

We found that p-AKT was elevated in a dose-dependent manner by SC79-mediated activation of AKT (Figures 6(b) and 6(h)). In response to 20 *μ*M SC79, HL-1 cells exhibited massive cell death. The expression of Rab27a also increased with increasing p-AKT levels (Figures 6(b) and 6(i)). Moreover, the expression levels of the downstream exosome markers CD81 and TSG101 were significantly increased (Figures 6(b), 6(j), and 6(l)). In contrast, GSK690693 significantly reduced the level of p-AKT (Figures 6(a) and 6(d)) and affected the expression of Rab27a (Figures 6(a) and 6(e)) and the downstream signaling pathway (Figures 6(a), 6(f), and 6(g)). Undoubtedly, the expression of exosome markers was significantly reduced by knocking down the expression of Rab27a (Figures 6(a), 6(g), and 6(f)). However, the knockdown of Rab27a significantly affected the expression of p-AKT (Figures 6(a) and 6(d)). Thus, we hypothesized that Rab27a could influence the expression of AKT through feedback regulation. Immunofluorescence analysis showed the effects of BAPTA, GSK690693, and si-Rab27a on the expression of the downstream effector molecule Rab27a. Compared with that in the pacing group, BAPTA, GSK690693, and si-Rab27a significantly reduced the fluorescence intensity of Rab27a (Figures 6(k) and 6(m)). These results demonstrated that intervening in the KCa3.1/AKT/Rab27a signaling pathway reduced exosome secretion.

### 3.8. KCNN4 Overexpression Affects the AKT/Rab27a Signaling Pathway

**Figure 7:**
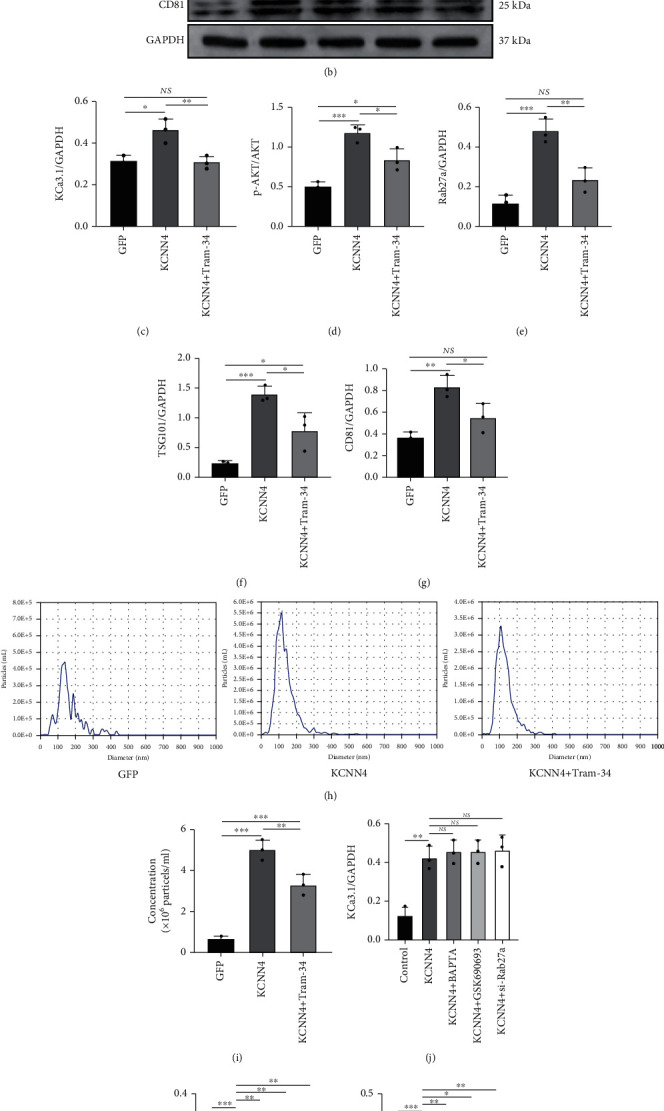
KCNN4 overexpression promotes exosome secretion. (a) Representative gel bands showing the effects of TRAM-34 administration on KCNN4-overexpressing HL-1 cell. (b) Representative gel bands showing the effects of BAPTA, GSK690693, and si-Rab27a on KCNN4-overexpressing HL-1 cell. (c–g). The levels of KCa3.1, p-AKT/AKT, Rab27a, TSG101, and CD81 in KCNN4-overexpressing HL-1 cell after TRAM-34 administration. (h, i) Mean exosome diameter and concentration, as shown by the Zeta View System. The exosomes were extracted from the same volume of supernatant from cells of equal density. (j–n) The levels of KCa3.1, p-AKT/AKT, Rab27a, TSG101, and CD81 in KCNN4-overexpressing HL-1 cell after treatment with BAPTA, GSK690693, and si-Rab27a. The data are presented as the mean ± SD of three biological replicates. ^∗^, ^∗∗^, and ^∗∗∗^ indicate *p* < 0.05, 0.01, and 0.001, respectively.

We constructed a stable KCNN4-overexpressing HL-1 cell line and found that cell proliferation was significantly increased, confirming the properties of the proto-oncogene of KCNN4. Compared with that in the KCNN4 group, TRAM-34 significantly inhibited the expression of KCa3.1 (Figures 7(a) and 7(c)). TRAM-34 also affected downstream signaling pathways to varying degrees. The expression levels of p-AKT (Figures 7(a) and 7(d)), Rab27a (Figures 7(a) and 7(e)), and exosome markers (Figures 7(a), 7(f), and 7(g)) were significantly decreased after the administration of TRAM-34. Moreover, nanoparticle tracking analysis demonstrated that the concentration of cellular supernatant exosomes increased to 4.9 ± 0.7 × 10^6^ particles/ml in the KCNN4 group, and this concentration was decreased to 3.3 ± 0.5 × 10^6^ particles/ml in response to TRAM-34 treatment (Figures 7(h) and 7(i)). Similar to the previous findings, calcium chelators, AKT inhibitors, and si-Rab27a did not alter KCa3.1 expression (Figures 7(b) and 7(j)) but significantly reduced the expression of p-AKT (Figures 7(b) and 7(k)), Rab27a (Figures 7(b) and 7(l)), and exosome markers (Figures 7(b), 7(m), and 7(n)). Therefore, our results showed that KCa3.1 overexpression increased exosome secretion through the AKT/Rab27a signaling pathway.

## 4. Discussion

This study examined the role of KCa3.1 in exosome secretion by atrial myocytes during rapid pacing in vivo and in vitro. We provide evidence that (1) KCa3.1 facilitates canine atrial structural remodeling during rapid atrial pacing. (2) KCa3.1 promotes exosome secretion by affecting the intracellular calcium concentration and activating the AKT/Rab27a signaling pathway. (3) These proinflammatory exosomes contribute to M1 macrophage polarization.

KCa3.1 channel phosphorylation and channel activity contribute to vital arrhythmogenesis in patients with arrhythmogenic right ventricular cardiomyopathy [16]. It has been reported that clotrimazole or TRAM-34 reduces the occurrence of catecholaminergic polymorphic ventricular tachycardia [7]. Our previous study showed that blocking KCa3.1 overexpression in canine atrial tissue macrophages using TRAM-34 reduced susceptibility to AF [9]. Recent studies have shown that the KCa3.1 channel allogenic blocker BA6b9 significantly prolonged the atrial and effective atrioventricular refractory periods in the isolated hearts of rats and reduced AF induction ex vivo [17]. This evidence supports the proarrhythmic role of KCa3.1. In contrast, Vigneault et al. reported that intramyocardial injection of human heart explant-derived cells and their extracellular vesicles after KCNN4 overexpression markedly increased cardiac function, the viability of the myocardium, and peri-infarct neovascularization [18]. These results indicated that KCa3.1 may play different roles in different spatial and temporal contexts.

Some studies have examined how ionic remodeling drives structural remodeling. Wang et al. reported that Ang II stimulated cell proliferation by upregulating the KCa3.1 channel through the ERK1/2, p38-MAPK, and PI3K/AKT signaling pathways in cultured adult rat cardiac fibroblasts [19]. Oxidative stress also promotes myocardial fibrosis by upregulating the KCa3.1 level [20]. In addition, KCa3.1 is overexpressed in diseases such as idiopathic pulmonary fibrosis [21], renal fibrosis [22], and postburn hypertrophic scar formation [23]. Using the specific blocker TRAM-34 inhibits the proliferation of fibroblasts and reduces the deposition of extracellular matrix by inhibiting TGF-*β*1 signaling [23]. These results showed that changes in ion channels have profound effects on cell proliferation and differentiation. The hyperproliferation and chemotaxis of fibroblasts and macrophages in myocardial tissue have an indelible effect on local structural remodeling. Structural remodeling provides an anatomical basis for reentry on the basis of ion remodeling by initiating factors.

The KCa3.1 channel is voltage-independent and Ca^2+^-sensitive, and its activation preserves the negative membrane potential required for sustained Ca^2+^ influx via store-operated Ca^2+^ channels. Abnormal Ca^2+^ handling promotes the secretion of exosomes. Olivero et al. found that the depolarization-induced release of exosomes from cortical synaptosomes occurred in a Ca^2+^-dependent fashion [24]. Interestingly, physical stimulation such as high-frequency acoustic stimulation also leads to calcium-dependent exosome release [25]. Our study showed that the calcium antagonist BAPTA reduced the expression of proteins related to exosome secretion. Thus, existing evidence supports an increase in exosome secretion due to calcium handling abnormalities in AF.

Current studies have shown that the overexpression of KCa3.1 promotes tumor cell proliferation and differentiation and may activate the PI3K/AKT signaling pathway mediated by calcium ions [26, 27]. Our study also verified that the overexpression of KCa3.1 led to HL-1 cell hyperproliferation. Excessive activation of AKT, which is the downstream signaling factor of KCa3.1, promotes fibrosis. Treatment of cardiac fibroblasts with Ang II promotes extracellular matrix deposition by upregulating PI3K/AKT expression [28]. In acetylcholine- and calcium chloride-induced mouse models of AF, AKT was also highly expressed in atrial tissue [29]. However, the degree of activation of the AKT signaling pathway in different cells is inconsistent. In contrast to the findings of Zou et al. [30], we found that overactivation of the AKT signaling pathway could be lethal to cardiomyocytes. SC79 (20 *μ*M) killed all HL-1 cells. Therefore, AKT overexpression may have adverse effects on cardiomyocytes to a certain extent.

To date, studies have not clearly defined how activation of the KCa3.1/AKT signaling pathway affects its downstream pathway and plays a regulatory role in cardiovascular disease. We found that the overexpression of KCa3.1 or rapid pacing enhanced exocrine functions. This effect was mainly reflected in the increased expression of Rab27a, which is a protein related to exosome secretion, and the increase in exosome markers. Interestingly, we found that TNF-*α*, IL-1, IL-6, and TGF-*β*1 levels in supernatants were significantly increased in the pacing group, which indicates that inferior stimulation may enhance cell secretion. This abnormal secretion may change the surrounding microenvironment, including local inflammation or fibrosis, and drive structural remodeling of atrial tissue.

Rab27a knockout impairs myeloperoxidase secretion by permeabilized neutrophils by interfering with the JFC1/Slp1-Rab27a signaling pathway [31]. Johnson et al. reported that AKT regulates JFC1/Slp1 function through phosphorylation and affects the secretion of Rab27a-containing vesicles [14]. Recent studies have shown that platelet-rich plasma promotes mesenchymal stem cell-derived exosomal paracrine repair of acute kidney injury via the AKT/Rab27 pathway [32]. These findings suggest that AKT is the upstream signaling factor of Rab27a. However, Zhou et al. found that Rab27a could affect the expression of AKT through feedback regulation by constructing Rab27a-knockout mice [33]. Consistent with their observations, our study confirmed that silencing Rab27a affected the phosphorylation of AKT.

The role of exosomes in macrophage polarization has been extensively studied. It has been reported that exosomes derived from inflammatory myoblasts or adipocytes promote M1 polarization [34, 35]. In addition to affecting cells in the surrounding microenvironment, exosomes also play a regulatory role through long-distance transport. Dasgupta et al. verified that the activation of IRE1A in hepatocytes resulted in ceramide biosynthesis and the release of exosomes. These proinflammatory exosomes recruited macrophages [36]. Broadly, our study suggests that TRAM-34 may reduce M1 polarization by blocking KCa3.1, which is involved in proinflammatory exosome release.

In conclusion, our study showed that KCa3.1 overexpression promoted proinflammatory exosome secretion through the AKT/Rab27a signaling pathway. Inhibiting the KCa3.1/AKT/Rab27a signaling pathway reduced myocardial tissue structural remodeling in AF. These results indicate a new therapeutic target for reducing the incidence of AF.

## Figures and Tables

**Table 1:** Primers.

	F: 5′-3′	R: 5′-3′
Canine		
iNOS	ACCAATACAGGCTCGTGCAG	GGGCTGTCTACTACTCGCTCC
Arg-1	GGCAGAAGTCAAGAAGAACGG	CTTTGGCAGATAGGCAAGGAG
IL-1*β*	ACCCGAACTCACCAGTGAAATG	GGTTCAGGTCTTGGCAGCAG
IL-6	CCCACCAGGAACGAAAGAGA	CTTGTGGAGAGGGAGTTCATAGC
GAPDH	GAAGGTCGGAGTGAACGGATT	CATTTGATGTTGGCGGGATC

Mouse		
IL-6	TGTGCAATGGCAATTCTGAT	GGTACTCCAGAAGACCAGAGGA
iNOS	GCTCATGACATCGACCAGAA	TGTTGCATTGGAAGTGAAGC
GAPDH	GAGAAGGCTGGGGCTCATTT	AGTGATGGCATGGACTGTGG

## Data Availability

All data involved in this study are available from the corresponding authors upon reasonable request.
